# A Velostat-Based Pressure-Sensitive Mat for Center-of-Pressure Measurements: A Preliminary Study

**DOI:** 10.3390/ijerph18115958

**Published:** 2021-06-02

**Authors:** Javier Martinez-Cesteros, Carlos Medrano-Sanchez, Inmaculada Plaza-Garcia, Raul Igual-Catalan, Sergio Albiol-Pérez

**Affiliations:** 1 EduQTech, E.U. Politécnica, University of Zaragoza, 44003 Teruel, Spain; ctmedra@unizar.es (C.M.-S.); inmap@unizar.es (I.P.-G.); rigual@unizar.es (R.I.-C.); salbiol@unizar.es (S.A.-P.); 2 Aragón Health Research Institute, IIS Aragón, University of Zaragoza, 50009 Zaragoza, Spain

**Keywords:** center of pressure, balance assessment, force platform, pressure sensitive mat, Velostat

## Abstract

Center-of-pressure (CoP) displacements play a key role in studies assessing postural stability. The accepted instrument to measure CoP trajectories is the force platform, but pressure-sensitive mats (PSMs) are an alternative composed of a matrix of sensitive cells. A typical cell comprises two electrodes with piezoresistive material in between, while a force platform has a force sensor at each of its corners. In this paper, we compare a homemade Velostat-based PSM and an affordable commercial mat with a commercial force platform in a test series with 42 healthy volunteers in single-legged trials (29 males, 13 females; height 1.74 (0.09) m, weight 74.3 (16.34) kg, age 31.21 (12.66) years). The aim of the research was to perform a preliminary study of the performance of our prototype to measure CoP, and more specifically, the standard deviation of the CoP path on both axes, the medial–lateral and anterior–posterior. We could thus discover several improvements for future clinical applications. The intraclass correlation coefficient (ICC) for agreement in the base experiment showed a moderate value for the prototype (0.38 to 0.63) and lower values for the commercial mat (0.11 to 0.12). However, we identified several factors that were relevant to improve ICC and reduce error by considering several processing options: (i) the known crosstalk problem between cells that appears in this kind of mats must be eliminated; (ii) the response time of the sensor has to be taken into account; and (iii) increasing the mat resolution also improves agreement. Therefore, as future work, we plan to test the improved version of the prototype in a clinical environment.

## 1. Introduction

Center-of-pressure (CoP) displacement is a widely accepted quantitative measure for stability studies in quiet standing. CoP is often measured using a force platform [1]. Thus, this device was extensively utilized in the literature in populations such as the elderly [2,3,4], infants [5], diabetic subjects [6], or children with cerebral palsy [7].

However, force platforms are usually expensive and cumbersome. Thus, many studies were conducted to find alternative instruments. A pressure-sensitive mat (PSM) is also an option to determine stability. A PSM covers relatively large areas, allowing for pressure to be measured in seats, beds, or on the floor. For instance, it was used to measure CoP and train sitting balance in patients who had had a stroke [8]. In this context, a PSM is more comfortable than a force plate is. Several studies compared commercial PSMs with force platforms. In [9], an AMTI^®^ force platform and a commercial plantar pressure system (Tekscan^®^ Strideway Plantar Pressure Mat) were used to measure the vertical component of ground reaction force in quiet-standing trials with eight weighting conditions. In [10], CoP measurements obtained from a MatScan^®^ PSM and AMTI^®^ AccuSway force platform were compared in single-legged balance trials with healthy volunteers. In [7], a Tekscan^®^ HR Mat and an AMTI^®^ force plate were used to assess standing balance in typical children and in children with cerebral palsy. In general, the studies found moderate-to-strong agreement between the instruments for some CoP parameters. However, CoP displacements derived from mats tended to be smaller compared with those measured with force plates. For instance, in [11], the authors concluded that the sway amplitudes for the PSM were slightly smaller. Examples were shown with a reduction of about 20–25% in the standard deviation of the CoP trajectory. In [10], the Bland–Altmann plots of the maximal CoP range also revealed smaller measurements for the mat, with averaged differences ranging from 0.14 to 0.42 cm, depending on the trial.

Nonetheless, commercial PSMs are usually expensive too. Therefore, there are many studies designing and developing new sensitive materials and configurations [12,13,14,15]. There are some low-cost pressure-sensitive materials readily available on the market: Velostat, Ex-Static^®^, or Eeon-Tex. For instance, Velostat was used in [14] to build a 32 × 32 sensor matrix for footprint evaluation. In [16], plantar foot data acquired by a self-made Velostat PSM were compared with a weight scale, the commercial Pedoscan platform, and the foot silhouette taken by ink on a paper. Even though the Velostat PSM had acquired better results than those of the commercial system in the weight scale, it could still not replace the foot silhouette. Other applications of Velostat-based PSM include monitoring neonatal constants [17], sport-training monitoring [18], and fall detection and elderly care [19].

The objective of this paper is to test the use of our first low-cost PSM prototype to measure CoP displacements in order to find its current limitations and promote future improvements. A Velostat-based PSM prototype and an affordable commercial PSM were compared with a force platform, which is the most widely accepted system for measuring CoP. Performance under different processing options is considered to reveal the influence of several factors. The long-term goal of our research is to use low-cost PSMs for balance studies. In the current paper, we present the first step towards this goal.

Previous similar studies considered only high-end commercial PSMs. However, each device is based on different patented technologies, mainly concerning the sensing material and manufacturing process. Thus, performance can largely vary, and there is still research being produced to find reliable flexible pressure sensors [12]. To the best of our knowledge, the current paper presents the first study that tests a Velostat-based PSM for CoP measurements. Velostat can be used to build PSMs far more cheaply than with current commercial products.

## 2. Materials and Methods

### 2.1. Volunteer Description

For the selection of the volunteers, we admitted subjects between 18 and 65 years old who were able to understand written and spoken Spanish. Informed consent was also a requirement. Volunteers with physical or medical problems that prevented or advised against standing on one or both feet were excluded. The general characteristics of the volunteers are shown in Table 1.

### 2.2. Hardware Description

**Figure 1:**
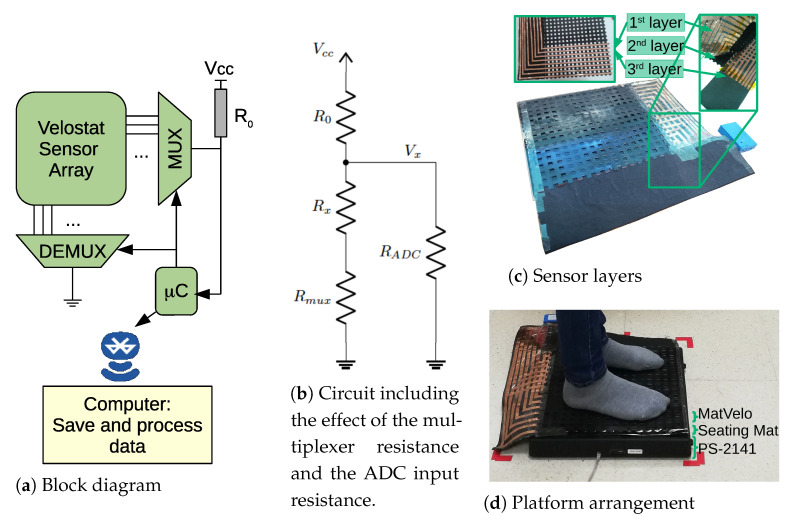
MatVelo PSM configuration and experiment setup with the commercial systems. (**a**) Block diagram of data-acquisition system; (**b**) equivalent circuit of each scanned cell; (**c**) layers of our custom-made PSM; (**d**) setup of the three overlapping platforms.

The homemade PSM prototype (MatVelo for short) was manufactured using commercially available materials (Figure 1). The system has three layers. The first layer consisted of a checkered flexible support built with a 3D printer. Conductive lines were formed using 7 mm wide adhesive copper tape, running vertically. The second layer was made of Velostat. Lastly, the third layer was similar to the first one, but with a rotation of 90°; conductive lines were, therefore, orthogonal on the first and third layers. The sensitive cells, of 7 × 7 mm, were located at the crossing points of the conductive lines. Overall, the mat comprised 16 × 16 sensitive cells covering 32 × 32 cm (Figure 1c).

The conductive lines were soldered to a connector to plug the data-acquisition system, which was enclosed in a plastic box. The electronic system is similar to those presented in previous studies [13,15]. A STM32F103C8T6 microcontroller scans rows and columns with analog multiplexers (Figure 1a). The selected row was connected to V_cc_ via an offset resistor, R_0_ = 2.2 kΩ, and to the microcontroller ADC. The selected column was connected to ground, thus forming a voltage divider circuit. However, the internal resistance of the multiplexers and the input impedance of the ADC were far from ideal when determining cell resistance. Therefore, they were calibrated and estimated as R_mux_ = 145 Ω and R_ADC_ = 123 kΩ. The equivalent circuit configuration used to find sensor resistance R_x_ from measured voltage V_x_ is shown in Figure 1b. The microcontroller was connected to a Bluetooth module that sends data to a PC. A program written in Processing was used to receive the data, store them in a file, and visualize the readings in real time for debugging. The whole mat could be sampled at 10 Hz. For the Velostat, conductance and pressure were approximately proportional [20,21].

We also tested a commercial PSM, Seating Mat by Sensing Tex [22]. According to the documentation, pressure sensitivity is obtained through a combination of electronic inks and pastes that are stretchable, elastic, and can be printed on materials with special surface preparations. The mat size and the number of spots of this product were the same as those in in our prototype. Each sensitive spot had a diameter of 10 mm, and pressure images were obtained at a frequency of 10 Hz. The conductance versus pressure curve of each spot is very close to a linear dependence. This commercial product is more affordable than many of its competitors. The software provided by the manufacturer was used to obtain raw data via Bluetooth. Then, we followed the documentation guidelines to convert them into conductance.

We also used a commercial force platform, the PS-2141 PASPORT Force Platform by Pasco [23]. Force platforms are considered as the gold standard for CoP measurements and they are used for the validation of instruments in this field. The PS-2141 has a load cell at each corner. This configuration makes it possible to obtain the vertical components of the ground reaction forces applied to the platform. Due to the rigidity of the platform, the load is redistributed proportionally to the four strain gauges in a 35-cm side square. The platform weighs 4 kg, its range is between −1100 N and 4400 N and its resolution is 0.1 N. The measurements were also taken at 10 Hz. The PASCO software records data via USB or Bluetooth, performs some processing and exports the data to a file.

Studies on the Velostat-based instrument are worth to be carried out because it has multiple advantages. It is a low-cost instrument with reduced complexity in the manufacturing process. Thus, it would be available for many research teams and medical centers. Moreover, it is flexible, lightweight, and transport-friendly, which allow for its use in irregular surfaces such as seats, backrests, and beds.

### 2.3. Protocol

The PSMs were laid on the force platform, one on top of the other (Figure 1d). The vertical order of the PSMs was randomly selected for each volunteer (in fact, we checked that there were no significant differences in the PSM outputs for the two possible positions). The systems were tested with humans to obtain the same kind of pressure images and irregular shapes of feet [14,24] as those in balance studies, which would be the ultimate target application; in this preliminary study, we only considered healthy subjects. Each participant performed the following eyes-open and barefoot trials on the platforms:

- Trial single-legged, right leg (RL): volunteers started the trial in single-legged quiet standing (flamingo posture, right leg). Then, they had to control their balance.
- Trial single-legged, left leg (LL): the previous trial was repeated, this time controlling their balance on the other leg (left leg).

A chair was placed close to the instruments to allow for volunteers to momentarily lean on its backrest if they were about to lose their balance. In this way, all the volunteers successfully carried out the tests, remaining single-legged throughout the entire tests.

We selected single-legged trials because they are very common [10,25]. As the selected volunteers were healthy, tests involving some difficulty should be performed in order to obtain relevant variations between subjects.

Each trial lasted 60 s, and the three instruments simultaneously acquired the data. However, the instruments were independently started, and the signals had to be aligned in a postprocessing step. To fully synchronize the signals coming from different instruments, we used crosscorrelation between them. This quantity is supposed to be maximal when the signals are fully aligned. Thus, the crosscorrelations of the Cartesian components of the CoP were obtained, and the signals were aligned to maximize them. The signals were displayed, and the initial time was corrected manually when required.

### 2.4. Data Processing

**Figure 2:**
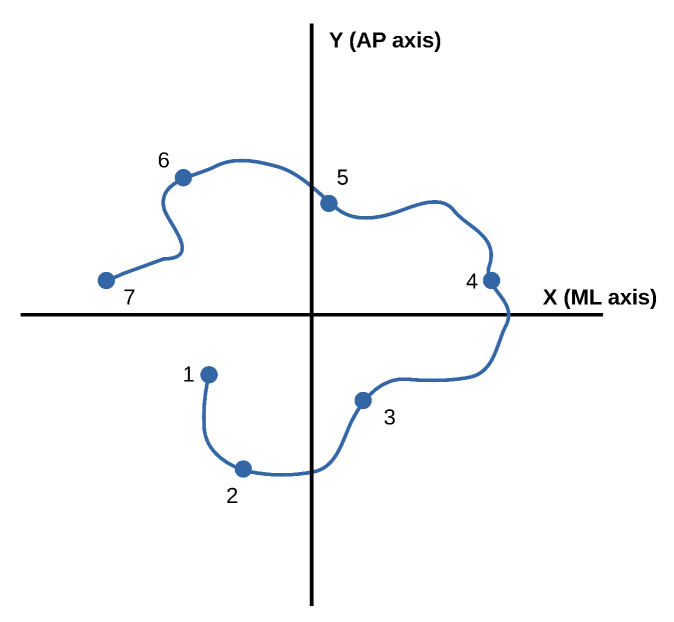
Diagram of CoP path, where X (ML) is the medial–lateral axis and Y (AP) is the anterior–posterior axis.

We focus on analyzing CoP excursion here. The CoP describes a trajectory over time, which makes it possible to detect tendencies in movements and potential health problems. Figure 2 presents a schematic representation of a CoP trajectory with seven captured points. More specifically, the standard deviation of the medial–lateral (ML) components of the CoP path, $\sigma_{\mathrm{ML}}$, and the standard deviation of the anterior–posterior (AP) components of the CoP path, $\sigma_{\mathrm{AP}}$, were extracted:

(1) $$\begin{array}{c} \sigma_{\mathrm{ML}}=\sqrt{\sum_{i=1}^{N}\frac{{\left(X_{i}-\bar{X}\right)}^{2}}{N}} \end{array}$$

(2) $$\begin{array}{c} \sigma_{\mathrm{AP}}=\sqrt{\sum_{i=1}^{N}\frac{{\left(Y_{i}-\bar{Y}\right)}^{2}}{N}} \end{array}$$

where $X_{i}$ and $Y_{i}$ are, respectively, the ML and AP components of the points in the trajectory, and N is the total number of captured points.

Thus, one value of $\sigma_{\mathrm{ML}}$ and one value of $\sigma_{\mathrm{AP}}$ were obtained for each subject, trial, and instrument. These values were the base for the device comparison. Analyzing the displacements on each axis is very common. In particular, standard deviation is a typical parameter of the CoP spread [26]. It is simple to calculate and less sensitive to outliers than the maximal range, for instance. Therefore, it is suitable for the current study of our first prototype. It was also used in many previous works [5,11,27] and shows good discrimination ability in different groups and pathology subjects [26].

**Figure 3:**
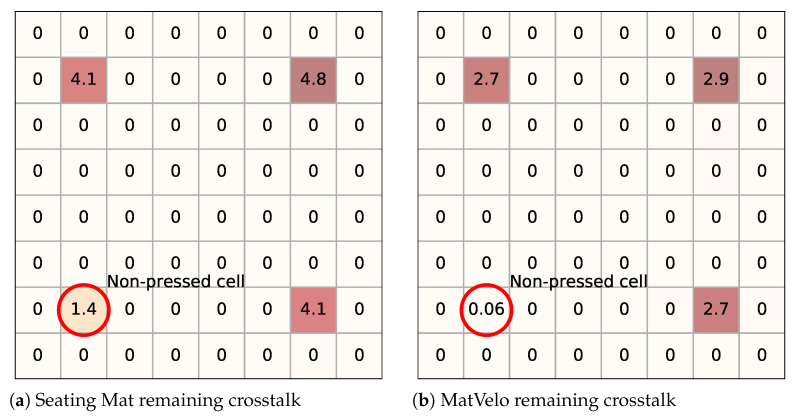
Excerpt of pressure images (arbitrary units) after elimination of crosstalk. (**a**) Seating Mat and (**b**) MatVelo remaining crosstalk.

The PS-2141 software directly returned the CoP coordinates. For PSMs, the CoP was obtained from the pressure measurements. However, PSMs made of resistive sensor arrays present a known crosstalk problem [28]: when addressing a single cell in the mat, the current flows both through that cell and through other cells in the array. Therefore, a row–column measurement gives the equivalent resistance of the mat between them, not the resistance of a single cell. This crosstalk is also called the “ghost effect” in the Seating Mat documentation [22] because it leads to pressure images with non-negligible values when there is no object on them. For instance, when pressing three cells at the corners of a rectangle, the fourth seems to also be pressed. Seating Mat software implements a correction for this effect. However, as Figure 3a shows, there is some residual value.

With respect to MatVelo, we applied the correction explained in [24,29]. The processing aims to invert the function that determines the equivalent resistance of a row–column measurement to obtain the values of each single cell. In this paper, the correction was further improved by taking into account the role of R_mux_ and R_ADC_, shown in Figure 1b, that is, for each row–column pair, the model in Figure 1b was used to obtain R_x_ from the measured voltage V_x_. Then, after a complete scan, the correction was applied as explained in [24]. The MatVelo result is shown in Figure 3b. In this case, the unpressed corner had negligible pressure after processing.

**Figure 4:**
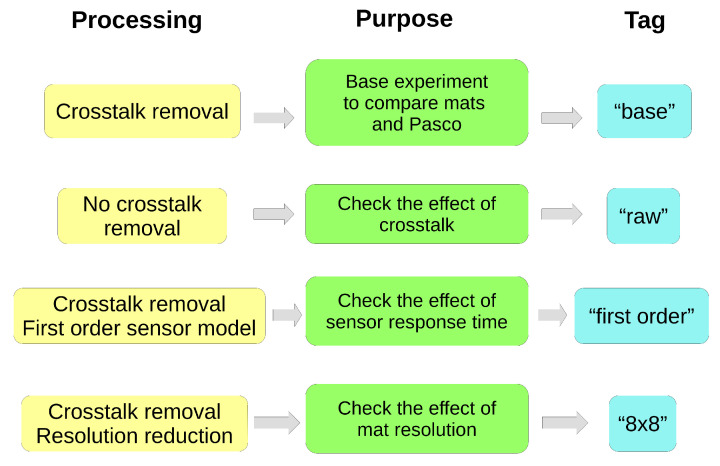
Schematic view of processing performed with MatVelo.

In addition, some variants of the basic processing for MatVelo were performed to study the effect of several limitations of the prototype. From now on, the processing explained in the previous paragraph is referred to as “base processing”. The other variants were derived from it and are described in the next subsections. They are also schematically shown in Figure 4.

#### 2.4.1. Variant Using Velostat Raw Data

Although the elimination of crosstalk is visually beneficial, it cannot yet be performed in real time and requires a postprocessing step. Therefore, we aimed to know whether it was relevant for the CoP. For that purpose, the role of the crosstalk elimination was studied by repeating analysis with the raw values of conductance without further processing. This condition is referred to as “raw” in this paper.

#### 2.4.2. Variant Using a First-Order Sensor Model

The nonideal response time of the sensor was taken into account. For this, a first-order system characterized by time constant τ was included. This variant is called “first order” here. A separate experiment with a single cell was performed to obtain τ by observing its exponential response after a pressure step input. The resulting value was τ = 0.25 s. Using this value, the nonideal response was included in the following way. First, we discretized the first-order system. Then, its equation was inverted, obtaining a typical finite impulse response filter of two coefficients [30]. Thus, the corrected value of pressure at time k, y[k], was obtained from the measured value of pressure at times k and k − 1, x[k] and x[k − 1], respectively:

(3) $$y\left[k\right]=\frac{1}{\left(1-a\right)}\cdot x\left[k\right]+\frac{a}{\left(a-1\right)}\cdot x\left[k-1\right]$$

with $a=e^{-T_{s}/\tau }$, being $T_{s}$ the sampling period.

#### 2.4.3. Variant with Resolution Reduction

Another aspect to be considered is the resolution of MatVelo. We emulated an experiment with an 8 × 8 mat by selecting only one out of two rows and columns of the original sensor matrix. The results corresponding to this variant are labeled as “8 × 8” in the paper.

### 2.5. Performance Evaluation

The comparison of the PSMs with the force platform was characterized using the intraclass correlation coefficient (ICC) for agreement [31]. ICC, which ranged from 0 to 1, estimates the ratio between true score variance and total variance taking into account the systematic differences between the two instruments and the random error variance. Moreover, since the CoP measurement based on force platforms is widely accepted, it can be considered to be a gold standard in this problem, with much less error than with measurements obtained from the mats. Thus, we also extracted the mean absolute error (MAE) considering the value returned by PS-2141 as the true value.

The above analyses were repeated for $\sigma_{\mathrm{ML}}$ and $\sigma_{\mathrm{AP}}$. ICC was calculated with R [32], while the processing of the raw data, and the calculation of CoP parameters and MAE were conducted in Python [33].

## 3. Results

The results for $\sigma_{\mathrm{ML}}$ and $\sigma_{\mathrm{AP}}$ averaged over volunteers in the two trials RL and LL are shown in Table 2. The obtained values from PSMs were lower than those producted with the force platform. This was even clearer for the Seating Mat, indicating that PSMs estimate a smaller trajectory.

The results of the comparison between the mats and the PS-2141 in the RL and LL trials are shown in Table 3 for ICC, and Table 4 for MAE. The ICC of the measurements using MatVelo was only moderate. The values depend on the specific experiment and population sample of young healthy subjects. ICC values did not reach 0.7, which is generally considered as the acceptable threshold [31]. The Seating Mat was even worse, with ICC values of around 0.1. Its error values were also worse.

**Figure 5:**
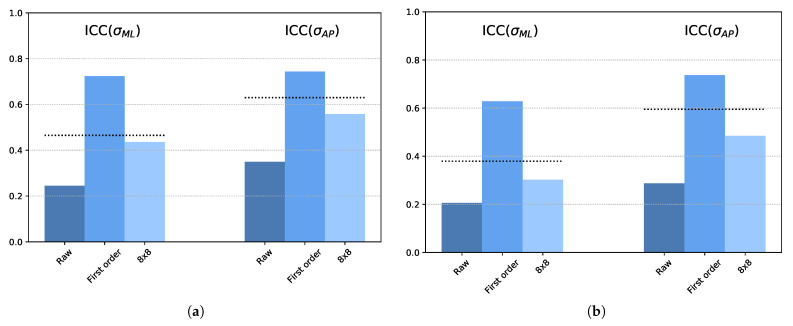

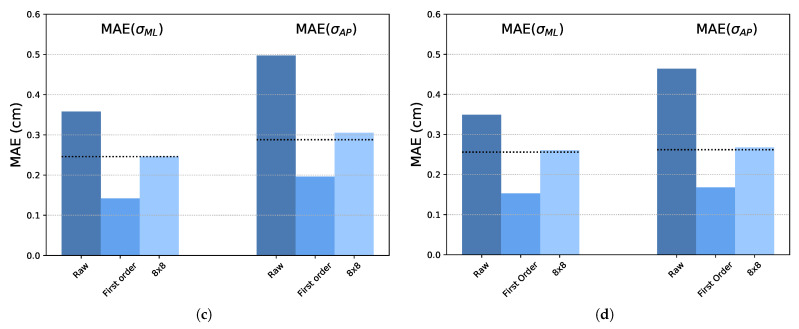
ICC and MAE for different processing conditions. (**a**) ICC, RL trial; (**b**) ICC, LL trial; (**c**) MAE, RL trial; (**d**) MAE, LL trial. Dotted horizontal lines, level of base processing for comparison purposes.

Figure 5 shows how different factors can alter ICC and MAE in MatVelo. The “base” processing results are included again as the horizontal dotted lines for comparison purposes. ML and AP displacements are included in the same figure. The “raw” processing option (taking raw sensor values without crosstalk correction) presented clearly worse results for all performance parameters. On the other hand, including the effect of the sensor response time and modeling it as a first-order dynamical system (“first-order”), led to an improvement in performance. In fact, the ICC = 0.7 level was reached except for $\sigma_{\mathrm{ML}}$ in the left-leg trials. In this particular case, it was higher than 0.6. With respect to resolution, a lower resolution (“8 × 8”) implied less agreement compared with the base experiment, as evidenced by the decrease in ICC. However, the effect on MAE in Figure 5 was less clear, with a small increase in error not always apparent.

## 4. Discussion

Experiment results indicate that the CoP derived from the Seating Mat was worse than the CoP derived from MatVelo. This was clearly due to the crosstalk removal. For the Seating Mat, we used a correction included in the manufacturer’s software. However, we conducted a simple test (for instance, pressing several single cells at the same time), and it visually appeared that the correction was not enough to completely remove the ghost effect, as explained in Section 2.4 and Figure 3. The worsening of the MatVelo performance if crosstalk was not removed also supported the importance of a correct elimination of this effect. Nonetheless, a problem of our current software implementation is that it is conducted in a postprocessing stage since it cannot reach real-time performance: typical processing time is around 0.1 s [24] at best, but additional time for data transfer and delays due to other PC tasks make it impossible to reach 10 Hz. This situation would be even worse with higher sampling rates and larger arrays.

The reasons for the low-to-moderate ICC values are extrinsic and intrinsic. On the one hand, all volunteers that participated in the experiment were young healthy control subjects. Thus, volunteer variance was not expected to be high. ICC would be zero for a perfect homogeneous sample. It is likely that measurements derived from MatVelo could explain more variance in the experiment if there were more clear differences between volunteers. Therefore, the current results indicate a rather lower limit for ICC, which would increase in a clinical environment. In that context, some people would present balance problems, and the variance of the sample would be higher. On the other hand, MatVelo presents some intrinsic limitations that are analyzed in the next paragraphs.

The effect of sensor response time was noticeable in the results, and taking it into account improved performance. Including it in the processing implies that the system acts as a low-pass filter. Thus, the output amplitude of a signal was lower than the input amplitude. This is in keeping with the differences shown in Table 2 in which the derived values from the force plate indicated a larger CoP spread. It is also in keeping with previous studies. In [10], a commercial pressure mat and a force plate were compared. Results showed a tendency to obtain smaller measurements with the mat when performing eyes-open and eyes-closed single-legged balance trials. The same effect was found in [11], which also compared a commercial mat and a force platform in quiet standing (eyes open and closed): in a particular case, the values for $\sigma_{\mathrm{ML}}$ were 1.49 and 1.98 mm, respectively, while for $\sigma_{\mathrm{AP}}$, they were 2.18 and 3.10 mm. Thus, it might happen that this is both a characteristic of our prototype and a common feature of many PSMs. A filter was also found in [11] to bring CoP trajectories of the PSM close to those of the force platform. The transfer function was numerically obtained in the frequency domain. This is similar to our idea of including the sensor response time and the associated first-order model.

With respect to resolution, our results showed its influence on performance. As expected, overall performance was worse for the lower resolution version, “8 × 8”. However, the MAE was almost the same, with a small increase not always noticeable in the figures. Digging into the data series, we realized that the most remarkable change was in the worst cases. This did not alter the mean error value shown in the figures too much; however, for instance, we checked that the 95th percentile was worse for the 8 × 8 version. High-end commercial PSMs present higher resolutions. For instance, in [10], the authors also considered healthy subjects, and one of the trials was single-legged eyes-open. In this test, strong correlations in the ML and AP ranges were found (Pearson’s r correlation coefficient = 0.93 and 0.99, respectively). They used a MatScan^®^ from TekScan that had 1.4 sensors/cm^2^, while MatVelo has 0.25 sensors/cm^2^.

The study presents several limitations. The considered population in this paper, young healthy volunteers, is different from the typical populations in which balance studies are usually performed. The trials may also be different because there is a large variety of test for balance measurements. For instance, the presented protocol cannot be used in stroke patients. Our results do not allow for drawing conclusions about different population segments or checking whether the device could detect pathologies. Thus, future research using the improved version of MatVelo should focus on a specific target population and balance test to reach the ultimate goal of a clinical application. The current paper is the first study to reveal some weak points of MatVelo. From an equipment point of view, homemade manufacturing has imposed some restrictions. The size of electrodes, number of cells, and distance between them were restricted by the typical size of copper tape and spacing suitable for manual manufacturing. Other regular technologies could be used to reach a better resolution without considerably increasing the cost, for instance, in flexible printed circuit boards.

To sum up the main findings of our study, we identified several improvements to implement in the next version of our prototype:

- Including a first-order model for the time response of Velostat or testing other low-cost materials (Ex-static or Eeon-Tex) for fast sensor responses.
- Increasing the mat resolution.
- Making the algorithm for crosstalk removal faster so that it could run in real time, even with larger arrays of sensors and higher sampling frequencies.

With regard to potential applications, several experiments could be performed. First, an experiment with both a control group and people with balance disorders could indicate the discriminative power of the mat despite its differences with the force platform. Second, an experiment in a clinical context involving older people with different degrees of balance control would indicate agreement in a real environment. It is expected that it would be higher in such a sample due to its diversity. Besides, we are going to test and analyze the CoP measured with MatVelo during a set of sessions using a system for rehabilitation developed in our group, NeuroVirt [34], in a novel study with stroke patients. Previous similar studies using a PSM [8] motivated us to look for our own affordable solutions.

The importance of the availability of equipment for balance studies cannot be underestimated. Stability analyses are performed every day. The use of a low-cost instrument would allow for quantitative measurements even in primary-healthcare centers, complementing doctor evaluations and controlling rehabilitation processes. The factors that we identified in the paper help to reach the goal of obtaining such an instrument. PSMs stand out for their portability, flexibility, and low weight, which makes them less difficult to use, transport, and store. A PSM of 41 × 41 cm can be rolled up to fit into a cylinder of ⌀ 3 × 41 cm, with a weight of 270 g (including electronics) compared to about 4 kg for a force platform.

## Figures and Tables

**Table 1:** Overall participant characteristics given as mean (SD).

	Men	Women	Total
No.	29	13	42
Height (m)	1.78 (0.07)	1.64 (0.06)	1.74 (0.09)
Weight (kg)	81.36 (14.21)	58.54 (7.21)	74.30 (16.34)
Age (years)	33.55 (12.47)	26.00 (11.91)	31.21 (12.66)

**Table 2:** $\sigma_{\mathrm{ML}}$ and $\sigma_{\mathrm{AP}}$ in cm for the two trials and three instruments, given as mean(SD), where $\sigma_{\mathrm{ML}}$ is the standard deviation of the medial–lateral (ML) components of the CoP, $\sigma_{\mathrm{AP}}$ is the standard deviation of the anterior–posterior (AP) components of the CoP. RL, right-leg trial; LL, left-leg trial.

	PS-2141		MatVelo		Seating Mat	
	RL	LL	RL	LL	RL	LL
$\sigma_{\mathrm{ML}}$	0.72 (0.18)	0.68 (0.16)	0.47 (0.16)	0.42 (0.14)	0.30 (0.13)	0.31 (0.10)
$\sigma_{\mathrm{AP}}$	1.13 (0.34)	1.01 (0.27)	0.84 (0.30)	0.76 (0.25)	0.37 (0.21)	0.32 (0.15)

**Table 3:** ICC of our PSM and the commercial PSM compared with the PS-2141, where ICC($\sigma_{\mathrm{ML}}$) and ICC($\sigma_{\mathrm{AP}}$) are the intraclass correlation coefficients of the standard deviation of the ML and AP components, respectively. RL, right-leg trial; LL, left-leg trial.

	MatVelo		Seating Mat	
	RL	LL	RL	LL
ICC($\sigma_{\mathrm{ML}}$)	0.46	0.38	0.12	0.11
ICC($\sigma_{\mathrm{AP}}$)	0.63	0.59	0.11	0.07

**Table 4:** MAE (cm) of our PSM and the commercial PSM, where MAE($\sigma_{\mathrm{ML}}$) and MAE($\sigma_{\mathrm{AP}}$) are the mean absolute errors of the standard deviation of the ML and AP components, respectively. RL, right-leg trial; LL, left-leg trial.

	MatVelo		Seating Mat	
	RL	LL	RL	LL
MAE($\sigma_{\mathrm{ML}}$)	0.25	0.26	0.42	0.37
MAE($\sigma_{\mathrm{AP}}$)	0.29	0.26	0.75	0.70

## Data Availability

PS-2141 data, raw pressure maps, and Python code to obtain COP paths, $\sigma_{\mathrm{ML}}$ and $\sigma_{\mathrm{AP}}$ were uploaded to IEEE Data Port, doi:10.21227/wh59-h632.

## Abbreviations

The following abbreviations are used in this manuscript:

COP	Center Of Pressure
PSM	Pressure Sensitive Mats
ICC	Intra-class Correlation Coefficient
RL	Right Leg
LL	Left Leg
ML	Medial-Lateral
AP	Anterior-Posterior
MAE	Mean Absolute Error
